# Construction and validation of a nomogram to predict overall survival in patients with breast sarcoma

**DOI:** 10.3389/fonc.2022.899018

**Published:** 2022-10-06

**Authors:** Yixin Cheng, Pengkun Zhang, Yulin Huang, Ru Tang, Lei Zhang, Jiayuan Sun, Feng Chi, San-Gang Wu, Zhenyu He

**Affiliations:** ^1^ Department of Radiation Oncology, Sun Yat-sen University Cancer Center, State Key Laboratory of Oncology in South China, Collaborative Innovation Center of Cancer Medicine, Guangzhou, China; ^2^ Department of Radiation Oncology, the First Affiliated Hospital of Xiamen University, Xiamen, China

**Keywords:** breast neoplasms, nomogram, sarcoma, prognosis, survival rate

## Abstract

**Background:**

This study aimed to construct a nomogram for Breast sarcoma (BS) to predict the prognosis of patients with BS accurately and provide a theoretical basis for individualized treatment.

**Methods:**

Patients selected from the Surveillance, Epidemiology and End Results (SEER) database from 2000 to 2018 were assigned to a training group (TG, n = 696) and an internal validation group (IVG, n = 299) at a 7:3 ratio. Cox regression analysis was performed on the TG, and statistically significant factors were used to establish a nomogram to predict 3-, 5-, and 10-year overall survival (OS). The nomogram’s predictive power was validated using data from patients who attended our institution as the external validation group (EVG, n =79).

**Results:**

Cox regression analysis identified five factors, which were used to construct the nomogram. Good prediction accuracy was demonstrated using calibration curves. The concordance (C) indices for TG = 0.804 (95% confidence interval (CI) 0.777–0.831) and IVG = 0.761 (0.716–0.806) were higher than those based on 8th American Joint Committee on Cancer (AJCC8) stage: TG = 0.695 (0.660–0.730), IVG = 0.637 (0.584–0.690). The EVG also had a high C-index: 0.844 (0.768–0.920). Decision curve analysis showed that nomogram has larger net benefits than the AJCC8. The Kaplan–Meier curves of the nomogram-based risk groups showed significant differences (p < 0.001).

**Conclusions:**

The nomogram could accurately predict 3-, 5-, and 10-year OS and provided nomogram-based risk stratification, which could help physicians to personalize treatment plans for patients with BS.

## Introduction

Breast sarcoma (BS) is a rare malignancy derived from mesenchymal cells, which can be divided into primary and secondary BS. Compared with the more common breast carcinoma, the natural history, treatment, and prognosis of BS are unique (1). According to clinical statistics, primary BS represents < 1% of all breast tumors and < 5% of all sarcomas (2). Research on BS is limited because of its rarity, and comprises mostly small retrospective studies and case studies; moreover, treatment strategies for BS are mostly based on the extrapolated research data from soft tissue sarcomas of the extremities (1). Currently, BS staging principally relies on the 8th American Joint Committee on Cancer (AJCC8) for soft tissue sarcomas. The staging system is based on tumor size (T), lymph node involvement (N), metastasis (M), and histological grade (3). However, studies have shown that age (4), histological type (1), and treatment all have different effects on the prognosis of BS.

A nomogram is a tool that predicts a certain clinical outcome or the probability of a certain event based on the values of multiple clinical indicators or biological parameters. It predicts the probability of a specific outcome for patients at any given time by incorporating a variety of disease indicators. Nomograms have the advantages of convenience, simplicity, accuracy, reliability, and practicality. They have been used widely to predict prognosis in various tumors, e.g., oropharyngeal carcinoma (5), breast cancer (6), and cervical cancer (7). However, to the best of our knowledge, a nomogram to predict the survival of patients with BS has not been reported.

Therefore, this study aimed to develop a nomogram that could predict the overall survival (OS) of patients with BS using data from the Surveillance, Epidemiology, and End Results database (SEER). Further SEER data was used for internal validation and data from patients attending the Cancer Center of Sun Yat-sen University (SYSUCC) were used for external verification. Ultimately, we aimed to provide a nomogram that could assist clinicians to accurately predict patient prognosis and that formed a theoretical foundation to formulated individualized clinical treatment strategies.

## Materials and methods

### Patient selection and study variables

Data for the analysis were obtained from the SEER database from 2000 to 2018 and from SYSUCC patients’ data from 2000 to 2015.

The inclusion criteria for patients from the SEER database were: (1) IDC-O-3, malignant = 8800/3-9581/3; (2) site record = breast; (3) first malignant primary indicator = yes. The exclusion criteria comprised: (1) Diagnosed at autopsy or from a death certificate; (2) Follow-up time < 1 month or the patient died within one month; (3) Lack of necessary information including ethnicity, tumor size, lymph node status, and surgery type. (4) Tumors with sizes < 0.5 cm and > 40 cm were also excluded (because of the low incidence of tumors in this size range) (3). Finally, the study included 995 patients.

Variables analyzed included ethnicity, sex, age at diagnosis, marital status, histological subtype, grade, laterality, tumor size, lymph node status, metastasis, axillary lymph node surgery (biopsy or dissection), surgery, radiotherapy, chemotherapy, and follow-up time. To adjust the nonlinear relationship between age and death risks, the best cutoff value of age was determined using the restricted cubic spline (RCS) with five knots. The best cutoff of tumor size was determined using X-tile (Yale School of medicine, New Haven, CT, USA). According to the above information, N stage and AJCC stage were determined according to AJCC8 staging standards. The endpoint of the study was overall survival (OS).

A total of 95 patients with BS attended SYSUCC from 2000 to 2015, and after following the same inclusion/exclusion criteria used for the SEER data, 79 of these patients were assigned to the external validation group (EVG).

### Nomogram development and risk stratification

The 995 patients from SEER were assigned randomly to the training group (TG, n = 696) and the internal validation group (IVG, n = 299) according to a ratio of 7:3. Univariate and multivariate Cox regression analyses were carried out on the TG data to identify statistically significant factors (P < 0.05). Next, backward stepwise regression was performed to calculate the Akaike information criterion (AIC): The lower the AIC value, the better the model (8). Finally, based on the selected variables, a nomogram to predict the OS of patients at 3, 5, and 10 years was constructed, and the IVG and EVG data were used to test the reliability and generalizability of the developed model. Based on the nomogram-derived scores of different variables, we calculated the total score for each patient. This allowed the patients to be assigned to three risk groups (low, middle, and high). X-tile was used to determine the optimal cut-off value of risk stratification, and for the three risk groups, Kaplan–Meier survival curves in the TG, IVG and EVG were drawn, respectively, and survival analysis was performed using the log-rank test.

The concordance index (C-index), receiver operating characteristic curve (ROC) analysis, and the area under the curve (AUC) of the nomogram and of the AJCC8 stage at 3, 5, and 10 years were calculated to assess the discriminative power of the nomogram. The correlation between the actual and predicted results was compared using calibration curves. After determining that the nomogram was accurate, the clinical utility of the nomogram was compared with that of AJCC8 using decision curve analysis (DCA).

All statistical analyses were performed using the R software v4.1.1. (https://www.R-project.org). P < 0.05 was considered to be a statistically significant difference.

## Results

### Patients characteristics

In the SEER database, after following strict inclusion and exclusion criteria, a total of 995 patients were included in the study and were randomized to the TG (n = 696) and IVG (n = 299). Cases from SYSUCC were classified as the EVG (n = 79). The median age at diagnosis of patients from the SEER database was 54 years old, (interquartile range (IQR), 43.5–64), the median tumor size was 4.6 cm (IQR 2.95–8), and the follow-up time was 81 months (range: 31–129 months). The median age at diagnosis, tumor size, and follow-up time of patients from the EVG were 43 years (37–53), 4 cm (3–7.5), and 84 months (34–128), respectively. The detailed demographic and disease characteristics of the patients are presented in **Table 1**.

**Table 1:** Baseline demographic and clinical characteristics of the patients.

Characteristics	SEER^a^		SYSUCC
	Training group (n = 696)	Internal validation group (n = 299)	External validation group (n = 79)
**Age (years)**			
< 43	159 (22.8)	72 (24.1)	37 (46.8)
≥ 43	537 (77.2)	227 (75.9)	42 (53.2)
**Sex (%)**			
Female	694 (99.7)	297 (99.3)	79 (100.0)
Male	2 (0.3)	2 (0.7)	0
**Ethnicity (%)**			
White	518 (74.4)	237 (79.3)	0
Black	94 (13.5)	36 (12.0)	0
Other	84 (12.1)	26 (8.7)	79 (100.0)
**Marital status(%)**			
Married	345 (49.6)	155 (51.9)	71 (89.9)
Unmarried	305 (43.8)	133 (44.5)	8 (10.1)
Unknown	46 (6.6)	11 (3.7)	0
**Histological subtype (%)**			
Malignant phyllodes tumor	393 (56.5)	167 (55.9)	55 (69.6)
Other	303 (43.5)	132 (44.2)	24 (30.4)
**Grade (%)**			
I	141 (20.3)	63 (21.1)	
II	134 (19.3)	52 (17.4)	
III	421 (60.5)	184 (61.5)	
**Laterality (%)**			
Left	360 (51.7)	146 (48.8)	39 (49.4)
Right	336 (48.3)	153 (51.2)	40 (50.6)
**Size (%)**			
< 42 mm	306 (44.0)	129 (43.1)	42 (53.2)
42–67 mm	143 (20.6)	71 (23.8)	23 (29.1)
≥ 67 mm	247 (35.5)	99 (33.1)	14 (17.7)
**Surgery (%)**			
Breast-conserving surgery	305 (43.8)	127 (42.5)	30 (38.0)
Mastectomy	382 (54.9)	166 (55.5)	48 (60.8)
No	9 (1.3)	6 (2.0)	1 (1.3)
**Radiotherapy (%)**			
No/Unknown	495 (71.1)	216 (72.2)	75 (94.9)
Yes	201 (28.9)	83 (27.8)	4 (5.1)
**Chemotherapy (%)**			
No/Unknown	559 (80.3)	241 (80.6)	58 (73.4)
Yes	137 (19.7)	58 (19.4)	21 (26.6)
**Axillary lymph node surgery (biopsy or dissection)**			
No	401 (57.6)	164 (54.9)	50 (63.3)
Yes	295 (42.4)	135 (45.2)	29 (36.7)
**AJCC8 (%)**			
I	140 (20.1)	62 (20.7)	
II	256 (36.8)	111 (37.1)	
III	237 (34.0)	102 (34.1)	
IV	63 (9.1)	24 (8.0)	
**N (%)**			
N0	654 (94.0)	280 (93.7)	73 (92.4)
N1	42 (6.0)	19 (6.4)	6 (7.6)
**M (%)**			
M0	668 (96.0)	293 (98.0)	68 (86.1)
M1	28 (4.0)	6 (2.0)	11 (13.9)

a SEER, Surveillance, Epidemiology and End Results database; SYSUCC, Cancer Center of Sun Yat-sen University; AJCC8, 8th American Joint Committee on Cancer; N, node; M, metastasis.

### Nomogram variable screening

Univariate and multivariate Cox proportional hazards regression analysis identified five independent prognostic factors for OS in the TG, comprising age at diagnosis, histological subtype, tumor size, surgery, and M stage. As calculated using stepwise regression, the five variables produced the smallest AIC value; therefore, there was no need to eliminate the variables. Full details are provided in **Table 2**.

**Table 2:** Analysis of overall survival using univariate and multivariate Cox analysis in the training set.

Characteristics	Univariate analysis		Multivariable cox	
	HR^a^ (95% CI)	P value	HR (95% CI)	P value
**Age (%)**				
< 43	1 (reference)			
≥ 43	1.66 (1.16–2.38)	0.006	2.81 (1.9–4.17)	**< 0.001**
**Sex (%)**				
Female	1 (reference)			
Male	1.57 (0.22–11.2)	0.653		
**Ethnicity (%)**				
Black	1 (reference)			
White	0.55 (0.39–0.78)	0.001	0.74 (0.51–1.09)	0.1249
Other	0.57 (0.35–0.94)	0.026	0.78 (0.46–1.3)	0.3315
**Marital status (%)**				
Married	1 (reference)			
Unmarried	1.79 (1.35–2.36)	< 0.001	1.27 (0.93–1.71)	0.128
Unknown	0.97 (0.52–1.82)	0.93	0.98 (0.52–1.86)	0.9538
**Histological subtype (%)**				
Other	1 (reference)			
Malignant phyllodes tumor	0.28 (0.21–0.38)	< 0.001	0.3 (0.21–0.42)	**< 0.001**
**Grade (%)**				
I	1 (reference)			
II	1.17 (0.69–1.99)	0.568	0.94(0.55-1.61)	0.8136
III	2.72 (1.8–4.12)	< 0.001	1.14 (0.72–1.78)	0.5778
**Laterality (%)**				
Left	1 (reference)			
Right	0.97 (0.74–1.27)	0.822		
**Size (%)**				
< 42 mm	1 (reference)			
42–67 mm	1.8 (1.2–2.7)	0.004	1.76 (1.15–2.69)	**< 0.001**
≥ 67 mm	3.85 (2.79–5.3)	< 0.001	3.03 (2.1–4.38)	**< 0.001**
**Surgery (%)**				
Breast-conserving surgery	1 (reference)			
Mastectomy	4.72 (3.33–6.69)	< 0.001	2.27 (1.51–3.4)	**< 0.001**
No	15.06 (6.72–33.74)	< 0.001	2.45 (1.02–5.9)	0.0459
**Radiotherapy (%)**				
No/Unknown	1 (reference)			
Yes	1.17 (0.87–1.55)	0.299		
**Chemotherapy (%)**				
No/Unknown	1 (reference)			
Yes	2.42 (1.81–3.23)	< 0.001	0.9 (0.63–1.3)	0.5821
**Axillary lymph node surgery (biopsy or dissection)**				
No	1 (reference)			
Yes	1.83 (1.4-2.39)	< 0.001	0.86 (0.62–1.17)	0.3319
**AJCC8 (%)**				
I	1 (reference)			
II	1.17 (0.73–1.87)	0.52		
III	2.76 (1.79–4.27)	< 0.001		
IV	7.95 (4.89–12.91)	< 0.001		
**N (%)**				
N0	1 (reference)			
N1	2.88 (1.89–4.37)	< 0.001	1.37 (0.85–2.2)	0.1915
**M (%)**				
M0	1 (reference)			
M1	15.08 (9.89–23)	< 0.001	7.21 (4.3–12.08)	**< 0.001**

a HR, hazard ratio; CI, confidence interval; AJCC8, 8th American Joint Committee on Cancer; N, node; M, metastasis.

The bold values mean statistically significant P value.

### Nomogram construction and validation

**Figure 1:**
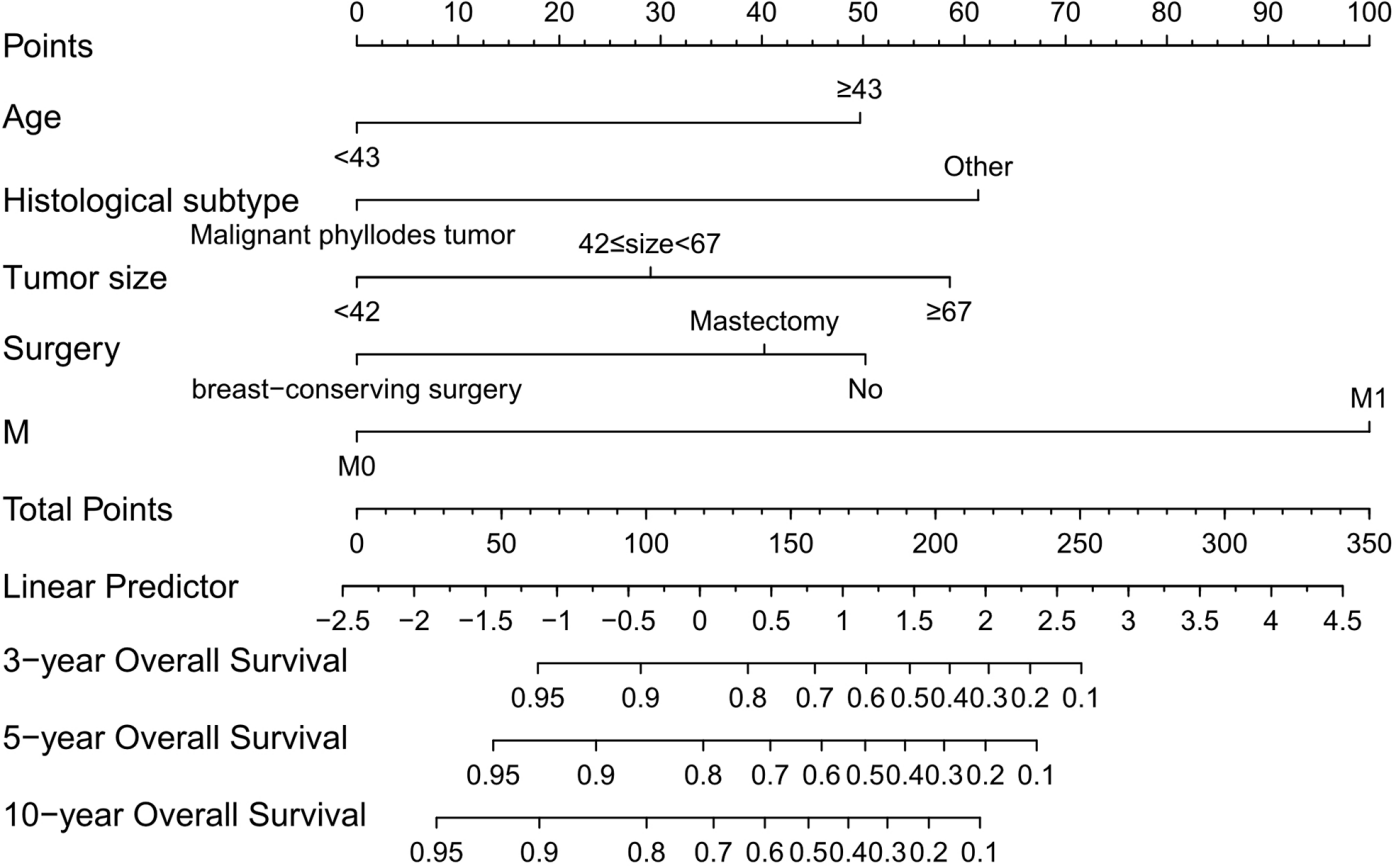
Nomogram that can predict the 3-, 5-, and 10-year overall survival of patients with primary breast sarcoma. M, metastasis.

Based on the screening results above, we established a nomogram to predict 3-, 5-, and 10-year OS (**Figure 1**). Each predictor was assigned a score, age < 43 years = 0 points; age ≥ 43 years = 50; malignant phyllodes tumor = 0; other histological subtype = 61; tumor size < 4.2 cm = 0; 4.2 ≤ tumor size < 6.7 cm = 29; tumor size ≥ 6.7 cm = 59; breast-conserving surgery = 0; mastectomy = 40; no surgery = 50; M0 stage = 0; M1 stage = 100. The scores for each variable were added to obtain the 3-, 5-, and 10-year OS probability of a patient.

**Figure 2:**
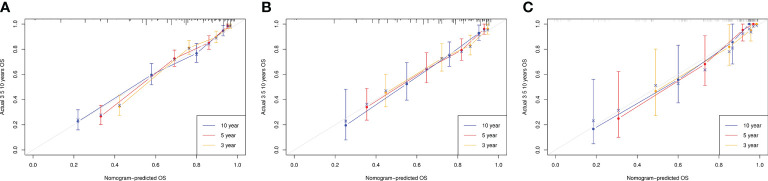
Calibration curves for overall survival (OS) at 3, 5, and 10 years in the training, internal-validation, and external-validation groups **(A–C)**.

The actual and predicted results showed good agreement based on calibration plots of the nomogram for 3-, 5-, and 10-year OS in the TG, IVG, and EVG (**Figure 2**).

**Figure 3:**
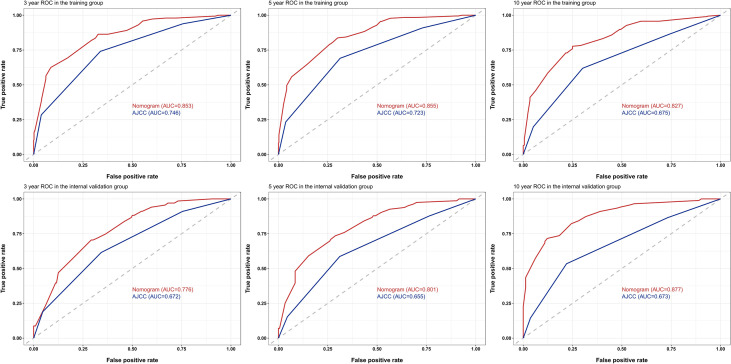
Receiver operating characteristic (ROC) curves for survival at 3, 5, and 10 years using the nomogram compared with AJCC8 data in the training and internal-validation groups. AUC, area under the curve; AJCC, American Joint Committee on Cancer.

**Figure 4:**
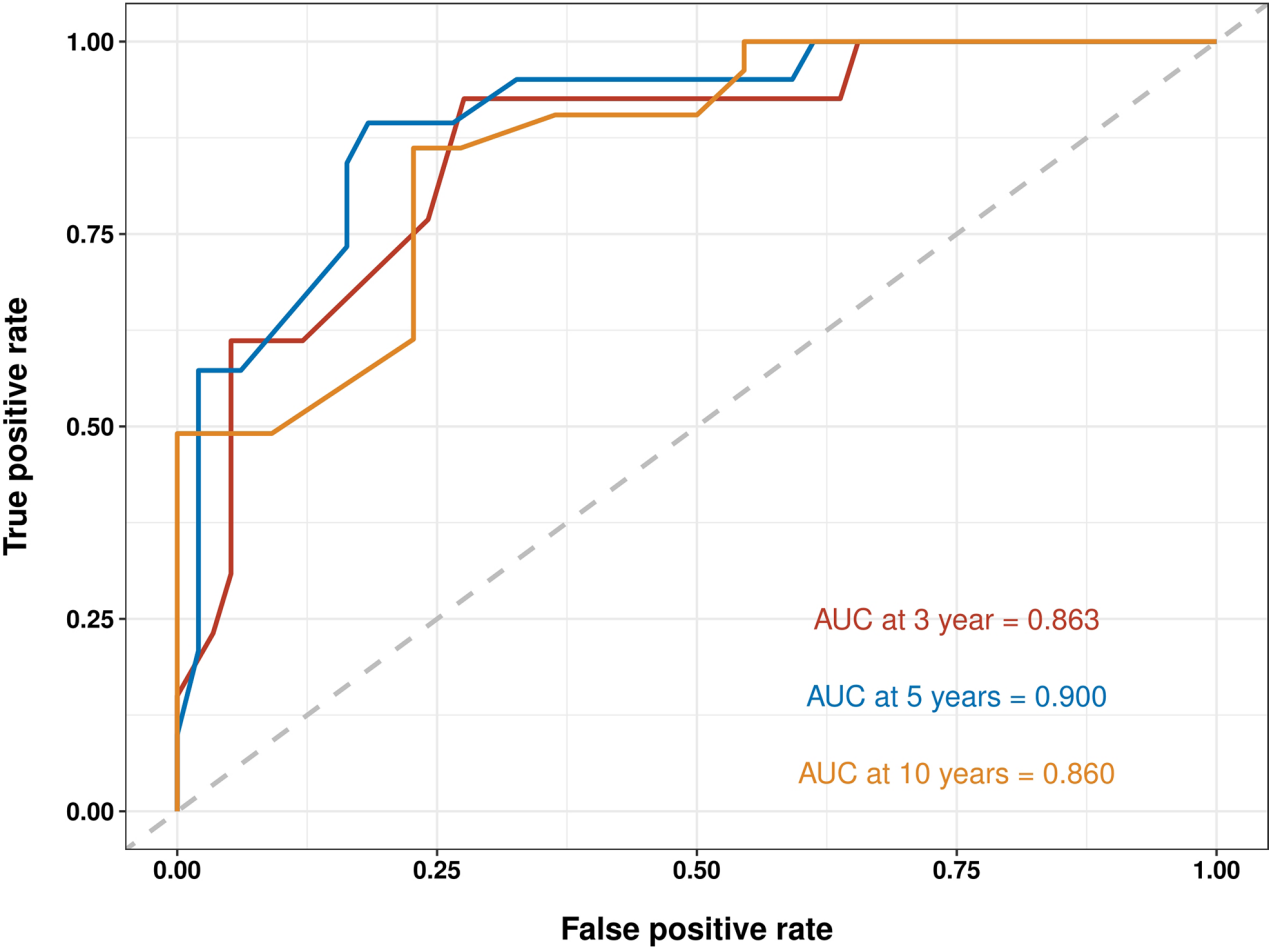
Receiver operating characteristic (ROC) curves for survival at 3, 5, and 10 years using nomogram in the external-validation group. AUC, area under the curve.

The C-indices to predict OS based on the nomogram were: TG = 0.804 (95% confidence interval (CI) 0.777–0.831) and IVG = 0.761 (95% CI 0.716–0.806), which were higher than those of the AJCC8 model: TG = 0.695 (95% CI 0.660–0.730), IVG = 0.637 (95% CI 0.5840.690), indicating the nomogram’s better discriminative ability. The results also showed an ideal predictive value in the EVG, with the C-index = 0.844 (95% CI 0.768–0.920). In addition, compared with the traditional AJCC8 staging model, the nomogram showed a better discrimination ability at different time points: the AUCs at 3, 5, and 10 years for the nomogram were 0.853, 0.855, and 0.827 in the TG; 0.776, 0.801, and 0.877 in the IVG. For the AJCC8 stage, the AUCs at 3, 5, and 10 years were 0.746, 0.723, and 0.675 in the TG; and 0.672, 0.655, and 0.673 in the IVG, respectively (**Figure 3**). These results suggested that the nomogram has a better power to predict OS than the AJCC8 stage. In addition, the AUCs at 3, 5, and 10 years for the nomogram to predict OS in the EVG were 0.863, 0.900, and 0.860, respectively (**Figure 4**), indicating that the nomogram model also has extremely high accuracy in practice (when AUC = [0.85, 0.95], the predicting effects are ideal).

**Figure 5:**
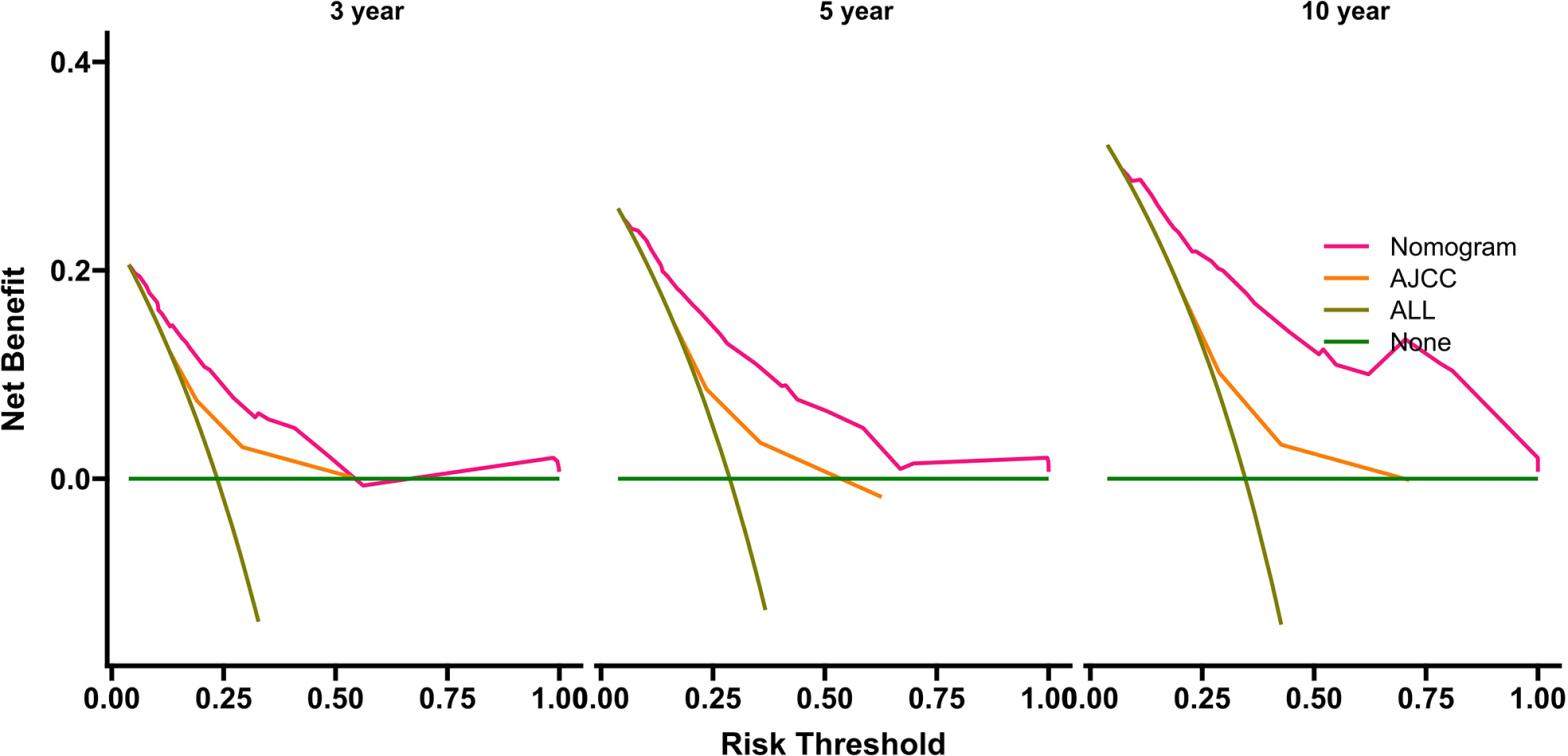
Decision curve analyses for survival at 3, 5, and 10 years using the nomogram compared with AJCC data in the internal-validation group. AJCC, American Joint Committee on Cancer.

The benefits and clinical utility and benefits of the nomogram and AJCC8 were compared using DCA, and the areas under the DCA of the nomogram at 3, 5, and 10 years were larger than those of the AJCC8 staging, indicating that the nomogram can obtain a higher net benefit than the AJCC8 model (**Figure 5**).

### Nomogram-based risk stratification

**Figure 6:**
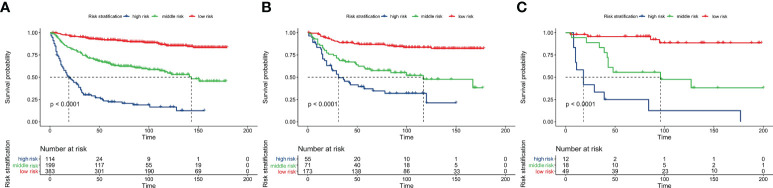
Overall survival analysis using Kaplan-Meier curves from the nomogram-based risk stratification in the training, internal-validation, and external-validation groups **(A–C)**.

The total score calculated by the nomogram was used for risk stratification: the best cut-off value was obtained by calculating the total score of all patients using the X-tile software. A patient’s probability of OS could be divided into three nomogram-based risk groups: 0 ≤ low risk < 130; 130 ≤ medium risk < 180; and high-risk ≥ 280. Kaplan–Meier survival curves for the three nomogram-based risk groups were then drawn. The new risk model displayed significant stratification power (p<0.001). To further validate the risk stratification ability of the nomogram, Kaplan–Meier survival curves for risk stratification in the IVG and EVG were drawn, and good results were obtained (p > 0.001) (see **Figure 6** and **Table 3** for details.)

**Table 3:** 3-, 5-, and 10-year overall survival in high-, intermediate-, and low-risk groups for each cohort.

		Training group	Internal validation group	External validation group
**high risk**	3-year survival	0.301	0.475	0.333
	5-year survival	0.222	0.371	0.250
	10-year survival	0.166	0.213	0.125
**middle risk**	3-year survival	0.741	0.686	0.833
	5-year survival	0.656	0.592	0.556
	10-year survival	0.546	0.477	0.476
**low risk**	3-year survival	0.945	0.888	0.957
	5-year survival	0.919	0.868	0.957
	10-year survival	0.855	0.835	0.886

## Discussion

Primary breast sarcoma is rare and has diverse histological differentiation; therefore, it is difficult to evaluate its prognosis. Currently, the principles of its diagnosis, treatment, and prognosis evaluation of malignancy mainly refer to soft tissue sarcoma occurring elsewhere in the body. Therefore, a reliable method to predict the prognosis of patients with primary BS and treatment guidance is urgently required. Currently, the most widely used tool to assess prognosis is the AJCC8 staging system; however, it lacks information on important predictors such as demographics and treatment. Nomograms are currently considered to be more scientific tools to predict prognosis; therefore, we constructed a nomogram to solve this clinical problem. To the best of our knowledge, this is the first study to construct a nomogram to predict the OS of patients with primary BS based on the big data SEER cohort, including validation of the accuracy of the model using retrospective data from an external single-center institution.

By analyzing variables such as demographics, clinicopathology, and treatment regimens, five variables were screened as predictors of OS, and the C-index, AUC, and DCA values of the nomogram were higher than those of the AJCC8 system, suggesting its superior discrimination ability and clinical significance. When using the nomogram to calculate the OS of EVG, it also showed a high C-index and AUC, demonstrating that the nomogram has high accuracy and practicability in predicting prognosis, and can help clinicians to personalize the evaluation of patient prognosis. Interestingly, over time, the AUC value and net benefit in the DCA of the nomogram increased compared with those of the AJCC8 system, suggesting the nomogram might useful to predict long-term survival. In addition, this study also calculated the total score of the five variables using the OS nomogram, used X-tile to calculate the best cut-off value, and then divided the patients’ probability of OS into three risk groups: high, medium, and low. Since high-risk patients may be at increased risk of tumor-related mortality, intense systemic therapy might be the preferred strategy for high-risk patients. This would aid clinicians to provide patients with personalized diagnosis and treatment methods and follow-up measures, which could avoid under- or over-treatment for patients with BS.

As with breast carcinoma of epithelial origin, the vast majority of patients with BS are female (9); however, patients with BS are generally younger (median 54 years old in SEER) and have larger tumors (median 4.6 cm in SEER) (10–12), and neither marital status nor ethnicity were independent prognostic factors (9, 13). Our study showed that age, tumor size, and metastasis are risk factors for OS in patients with BS, which is similar to other studies of sarcoma (12, 14–17). In addition, in this study, histological grade, although statistically significant in the univariate Cox analysis, was excluded in the multivariate Cox regression analysis (p ≥ 0.05), which was different from other studies (2, 12, 18, 19). Similarly, most previous studies reported that malignant phyllodes tumor (MPT) accounted for the most cases in BS, and in this study, MPT comprised 55.6% of cases in the TG and 69.6% in the EVG; angiosarcoma was the highest histological subtype after MPT (9% in the SEER database) (1, 20).

Although nearly half of the patients underwent axillary lymph node biopsy or dissection (43.2% in the SEER database and 36.7% in the EVG), our study showed that lymph node involvement was not an independent prognostic factor for OS, possibly because BS mainly metastasizes through blood, but rarely through lymph nodes. While most of the lymphadenopathy in clinical practice is reactive, lymph node dissection does not bring survival benefits to patients with BS (1, 11, 12, 21). Interestingly, although mastectomy is widely regarded as the gold standard for BS treatment (11), our study showed that patients receiving mastectomy did not have a better prognosis than those receiving conservative surgery, which was consistent with recent reports (1, 10, 14, 22). Therefore, more prospective studies are needed to explore whether mastectomy can provide additional benefits for patients with BS Studies suggest that adequate resection is an important factor for the long-term survival of patients, rather than the surgical method (1, 2, 23–26). In addition, like most studies (1, 27, 28), this study suggested that neither radiotherapy nor chemotherapy, or other adjuvant treatments, could significantly improve the OS of the patients; however, a study by Johnstone et al. showed that postoperative radiotherapy can improve the tumor specific survival of patients (20). Therefore, adjuvant radiotherapy is recommended for patients with BS with a large tumor volume or a particularly aggressive subtype (19, 29). Given the divergence in adjuvant therapy for BS, more prospective studies are needed to explore whether adjuvant therapy can improve patient outcomes.

The following limitations of the study need to be pointed out. First, although we randomly divided the SEER data into the TG and IVG and used an external data cohort as the EVG for validation, larger multicenter data are needed to confirm the results of the study. Second, some the SEER database lacked potentially important parameters and specific information associated with prognosis, including chemotherapy, radiotherapy, vascular invasion, and details of the surgical margin status. These important variables need to be considered in future studies. Furthermore, although research has demonstrated that histological grade is not an independent risk factor for the construction of nomograms, this information is lacking in the EVG patient data. In addition, the study was retrospective in nature and was thus inevitably biased.

## Conclusion

In this study, we constructed and validated a nomogram to predict OS in patients with BS, the reliability and clinical applicability of which were higher than those of the currently used AJCC8 staging system. We also developed a universal risk stratification model for clinical use, which provides clinicians with an accurate prognostic evaluation tool and will help to formulate the best individualized treatment strategy for patients with BS.

## Data availability statement

The raw data supporting the conclusions of this article will be made available by the authors, without undue reservation.

## Ethics statement

The studies involving human participants were reviewed and approved by the hospital ethics committee at Sun Yat-sen University Cancer Center. Written informed consent for participation was not required for this study in accordance with the national legislation and the institutional requirements.

## Author contributions

YC and PZ designed the study. YH and JS drafted the manuscript. RT, FC, and LZ analyzed the data. S-GW and ZH were responsible for the critical revision. All authors contributed to the article and approved the submitted version.

## Funding

This study was supported by the National Natural Science Foundation of China (No. 81872459), the Natural Science Foundation of Guangdong Province (No. 2018A030313666) and the Guangdong Medical Science and Technology Research Fund A2020516.

## Conflict of interest

The authors declare that the research was conducted in the absence of any commercial or financial relationships that could be construed as a potential conflict of interest.

## Publisher’s note

All claims expressed in this article are solely those of the authors and do not necessarily represent those of their affiliated organizations, or those of the publisher, the editors and the reviewers. Any product that may be evaluated in this article, or claim that may be made by its manufacturer, is not guaranteed or endorsed by the publisher.
